# Balancing conflict and coexistence: Interactions between invasive monk parakeets and native urban birds

**DOI:** 10.1002/eap.70275

**Published:** 2026-06-18

**Authors:** Jon Blanco‐González, Isabel López‐Rull, Fernando Enríquez, Luis Cayuela

**Affiliations:** ^1^ Área de Biodiversidad y Conservación, Universidad Rey Juan Carlos Móstoles Spain; ^2^ Instituto de Investigación en Cambio Global, Universidad Rey Juan Carlos Móstoles Spain; ^3^ Mantenimiento de Infraestructuras S.A.U. (MATINSA) Madrid Spain

**Keywords:** aggression, commensalism, competition, facilitation, intimidation, invasive species, monk parakeet, sparrow

## Abstract

Biological invasions often generate complex ecological paradoxes, particularly when invasive species act as ecosystem engineers that simultaneously compete with and benefit native communities. Understanding these dual dynamics is critical for managing urban biodiversity. Here, we investigated the interactions between invasive monk parakeets (*Myiopsitta monachus*) and native avifauna to assess the balance between behavioral competition and structural commensalism. We assessed competition through (1) agonistic interactions and (2) correlations between parakeet abundance and that of native species. Commensalism was evaluated by analyzing tenant species in parakeet nests and the drivers of their occurrence. Agonistic interactions manifested through highly species‐specific conflicts: Density‐dependent aggression with rock pigeons (*Columba livia*) was strictly reciprocal, while parakeets directed targeted intimidation toward Eurasian magpies (*Pica pica*). Conversely, direct agonistic encounters involving either Eurasian tree sparrows (*Passer montanus*) or house sparrows (*Passer domesticus*) were negligible. However, spatially, *Passer* spp. abundance correlated negatively with the number of parakeet nest chambers (a proxy for parakeet abundance), whereas common blackbirds (*Turdus merula*) showed a positive correlation. Furthermore, parakeets provided a massive structural subsidy. We recorded 11 native species breeding in 48% of surveyed parakeet nests (*N* = 252). Tree sparrows and stock doves (*Columba oenas*) dominated this tenant community, accounting for 86% of native breeding pairs. Native breeding abundance—including tree sparrows, stock doves, and rock pigeons—as well as total species richness scaled positively with nest chamber density. Yet, active parakeet presence limited nest use for stock doves but did not deter tree sparrows or rock pigeons from successful co‐nesting. Our findings reveal a dual ecological dynamic: Parakeets show a negative spatial correlation with declining urban sparrows, yet simultaneously act as ecosystem engineers by providing valuable breeding habitats for local biodiversity. However, without data on tenant reproductive success and pathogen transmission, these novel subsidies risk functioning as ecological traps. Consequently, while indiscriminate nest removal could inadvertently harm native tenants, current evidence does not confirm the long‐term safety of this commensalism. Effective management must transcend simple eradication, adopting a holistic framework that weighs the loss of nesting resources against the competitive and sanitary risks of retaining these invasive populations.

## INTRODUCTION

Biological invasions are a major environmental concern due to their widespread impact on biodiversity (Shochat et al., 2010). Avian species represent a significant component of global biological invasions, with approximately 400 species introduced around the world (Kumschick et al., 2016). Introduced bird species can impact native biota across ecological levels. At the population and community levels, effects include hybridization, resource competition, disease transmission, predation, and changes in herbivory patterns (Baker et al., 2014). At the ecosystem level, bird introductions can increase nutrient input into water bodies via droppings—potentially causing eutrophication—and alter plant and animal communities by establishing new interspecific interactions such as mutualisms (e.g., pollination, seed dispersal) or commensalism (e.g., nest facilitation) (Baker et al., 2014; Hernández‐Brito et al., 2021; Richardson et al., 2000).

While the major impacts of certain invasive bird species are well documented (e.g., Barilani et al., 2007; Pell & Tidemann, 1997; Van Riper et al., 1986), data remain scarce for three‐quarters of the regions they inhabit (Evans & Blackburn, 2020). Moreover, their broader ecological consequences are less studied compared to other taxa (but see Kumschick & Nentwig, 2010; Kumschick et al., 2015; Martin‐Albarracin et al., 2015; Shirley & Kark, 2009). This knowledge gap, combined with the complexity of native–invasive interactions—which can include commensalism (e.g., nest facilitation)—contributes to the persistent debate on whether eradication efforts are necessary (e.g., Strubbe et al., 2011). Therefore, to prioritize management efforts, it is essential to expand our knowledge on invasive bird species with the highest potential to negatively impact native biodiversity (Evans & Blackburn, 2020).

Species exhibiting the greatest overall impacts tend to be habitat generalists, multibrooded, smaller bodied, and prone to forming large feeding or roosting flocks (Martin‐Albarracin et al., 2015; Shirley & Kark, 2009). Several invasive species within the family Psittacidae display these characteristics. In fact, this family is recognized as having one of the highest impacts on European native ecosystems and the largest economic impact among invasive bird families (Kumschick et al., 2016; Kumschick & Nentwig, 2010). Ecological impacts of psittacine species are documented for 72% of successfully introduced species and include disease transmission to native wildlife and competition (Kumschick et al., 2016; Shirley & Kark, 2009).

One of the most successful psittacine invaders is the monk parakeet (*Myiopsitta monachus*, Boddaert 1783). Originally from South America, this species has established populations in 26 countries due to the pet trade (Calzada Preston & Pruett‐Jones, 2021), with individuals escaping or being deliberately released, particularly in urban areas (Da Silva et al., 2010). Spain currently hosts Europe's largest population, with an estimated 21,000 individuals in 2015, 40% of them in Madrid (Molina et al., 2016).

So far, research on the ecological impacts of monk parakeets has largely focused on disease transmission, particularly their role as vectors of parasites and pathogens affecting native birds (e.g., Aramburú et al., 2003; Blanco‐González et al., 2024; López et al., 2023; Mori et al., 2019). Regarding competition, studies are limited and often anecdotal (e.g., Batllori & Nos, 1985; Martella & Bucher, 1984; Wagner, 2012) with only a few of them providing detailed analysis of agonistic interactions between monk parakeets and native species (Briceño et al., 2019; Di Santo et al., 2016). These studies suggest that monk parakeets are not consistently more aggressive than co‐occurring native species, but they did not explicitly examine how intimidation or displacement varies with the body size or trophic similarity of the interacting species. In other bird assemblages, in fact, interspecific dominance and displacement at shared food resources are strongly structured by body mass and foraging niche, with larger, trophically similar species routinely displacing smaller ones from feeding sites (Dhondt, 2012; Miller et al., 2017). Moreover, equally overlooked are commensal interactions involving monk parakeets, even though this species has been described as an “ecosystem engineer” providing potential nesting substrates for cavity‐nesting birds (Briceño et al., 2019; Hernández‐Brito et al., 2021).

The monk parakeet is classified as an invasive alien species within the Spanish Catalog (Royal Decree 630/2013), legally obligating competent authorities to implement control, management, and potential eradication measures. However, the proposed interventions, which include lethal population control, frequently elicit social conflict. This issue is further exacerbated by the limited conclusive evidence demonstrating significant negative impacts of invasive monk parakeets on native biota. Moreover, the aforementioned commensal nesting dynamics further increase this ecological complexity, contributing to societal polarization and hindering effective management strategies. Under this scenario of rapid population growth (Senar et al., 2019), a better understanding of the net ecological effects of monk parakeets in urban areas is essential to contribute to the broader knowledge of avian invasions and provide a framework for evidence‐based management decisions. Here, we quantify both competitive and commensal pathways by which monk parakeets may affect native urban birds, using Madrid as a case study. Specifically, we assessed competition by (1) quantifying agonistic interactions between monk parakeets and native species during a foraging experiment, and (2) relating monk parakeet density to the abundance of three native bird taxa. To analyze commensalism, we examined nest‐site use by recording the abundance and species richness of tenant species breeding in monk parakeet nests and the factors influencing their occurrence. We conducted these evaluations based on three hypotheses. First, considering foraging niche overlap, we hypothesized that dominance during competition between monk parakeets and native species is determined by body size. Accordingly, we predicted that monk parakeets would dominate interactions with smaller native species, while being subordinate to larger ones. Second, we hypothesized that spatial displacement and abundance variations are driven by the degree of ecological similarity. We therefore predicted that increasing monk parakeet density would be more tightly associated with reductions in the abundance of smaller species with higher niche overlap than with that of larger species with more divergent dietary habits. Finally, we hypothesized that monk parakeets act as ecosystem engineers by providing novel structural resources that create opportunities for native breeding. Therefore, we predicted commensal nesting, with both the number of native pairs and native species richness increasing with the number of available nest chambers.

## METHODS

### Study area

**FIGURE 1:**
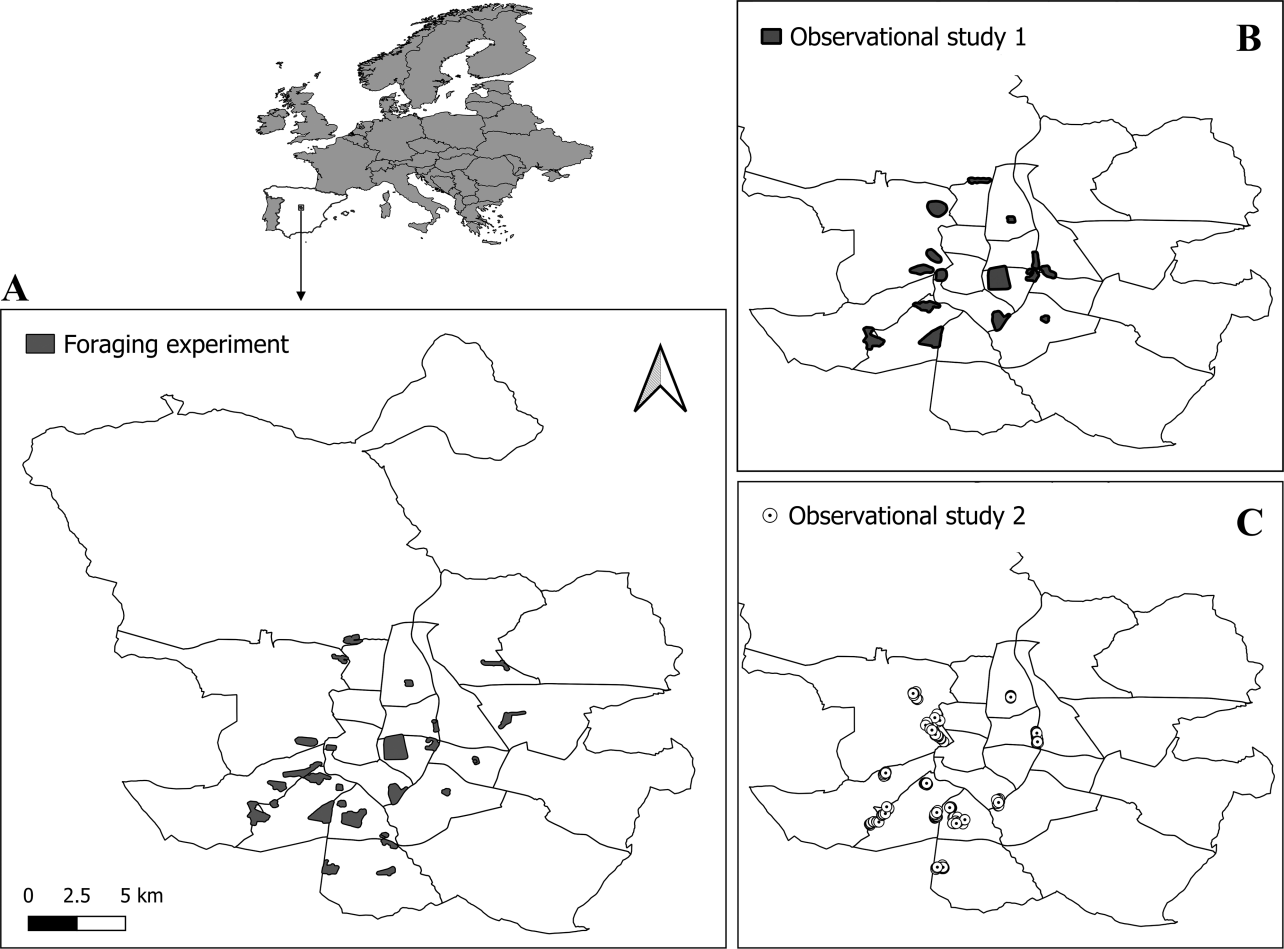
Study sites in Madrid, Spain. (A) Foraging experiment: Locations where aggressive and intimidating interactions among monk parakeets and native birds were recorded at feeding stations. (B) Observational study 1: Locations where monk parakeet, sparrow, and blackbird abundances were recorded. (C) Observational study 2: Locations where monk parakeet nests were examined to identify the use of parakeet nests by native species.

The study was conducted in the city of Madrid, Spain (Figure 1), which covers 604 km^2^ and lies at an average altitude of 657 m above sea level (asl). The city has a continental Mediterranean climate, with mild, wet winters and warm, dry summers. The monk parakeet population in Madrid was subjected to a management program between May 2021 and April 2023. The plan reduced the projected 2023 population by 50% (Blanco‐González et al., 2025). However, following the cessation of control efforts, the population was expected to recover to pre‐control levels by 2025, with an estimated 18,000 reproductive individuals (Blanco‐González et al., 2025).

### Foraging experiment: Agonistic interactions between monk parakeets and native birds

The study was conducted from 26 January to 21 March 2023 in 27 parks located within the city of Madrid, all with confirmed monk parakeet presence (Figure 1A). A total of 30 replicates for the foraging experiment were performed across 27 distinct park locations. Of this total, 24 trials were conducted once in 24 separate parks. The remaining six trials were carried out in three parks, each park receiving two trials conducted at different locations within the park and separated by an interval of at least 12 days. These three parks were selected for replication because they were the only available locations that strictly met all experimental requirements, including confirmed parakeet presence and sufficient open space to maintain identical spatial configurations for the experimental setup. In each trial, the setup consisted of four feeding stations (hereafter, “feeders”), each measuring 1 × 1 m and placed on the ground. All feeders were positioned in areas with similar spatial features (e.g., tree cover, distance to pathways) and contained 1 kg of bait evenly distributed to enhance visibility. Each feeder contained a different bait (stale bread, apple, commercial parrot feed, or a mixture of these), tested as part of a broader study evaluating their effectiveness in attracting parakeets (Blanco‐González et al., 2025). No refills were provided during the experiment. Feeders were arranged in a cross pattern with 90° angles between adjacent feeders, approximately 20 m from the observers. Their relative positions were randomized daily (Appendix S1: Figure S1).

A total of 120 behavioral observations were conducted (30 trials × 4 feeders) half an hour after sunrise. Each day, two observers were positioned at the center of the cross and recorded data for 2 h, starting when the first monk parakeet arrived at any of the feeders. Each observer monitored simultaneously two adjacent feeders. The observations consisted of scan sampling in 5‐min intervals, recording for each interval: (1) the number of monk parakeets and native bird species visiting the feeders (each individual was counted upon entry, regardless of previous visits); (2) the number of intra‐ and interspecific aggressions; and (3) the occurrence (1‐0 recording) of intra‐ and interspecific intimidations. Both the species initiating and receiving aggression or intimidation were identified. To minimize observer bias, observers switched feeders every 5 min. Aggression was considered when there was a direct attack—either with or without physical contact—of one bird toward another. Intimidation was considered when a bird showed subordinate behavior in response to the mere presence of another bird. Subordinate behavior was considered to occur in any of the following scenarios: (1) a bird at the feeder fled upon another bird's arrival (regardless of later return); (2) a bird showed interest in the feeder but remained on its periphery while others fed; (3) a bird moved to another part of the feeder when another bird approached; or (4) a bird was startled by an aggression between other birds, eliciting a response similar to the previous scenarios. Aggressions were quantified in 5‐min intervals, whereas intimidations were recorded only as present or absent due to the challenge of precisely counting individuals involved in intimidation events. We restricted our analysis of interactions to the four most frequent native visitors: Eurasian magpie (*Pica pica*), rock pigeon (*Columba livia*), Eurasian tree sparrow (*Passer montanus*), and house sparrow (*Passer domesticus*), as visits by other species were too sporadic to allow for robust statistical modeling.

### Observational study 1: Correlations between monk parakeet and native bird abundances

Fieldwork was conducted during three consecutive spring seasons (March–May) from 2021 to 2023 across 15 parks with confirmed monk parakeet presence (Figure 1B). In each park, line transects of approximately 200 m in length (mean = 181 m, SD = 42 m) were established to ensure representative coverage while accounting for habitat heterogeneity. The number of transects was proportional to park size, ranging from 5 to 10 per park, and all were positioned to avoid overlapping observation areas. Each transect was sampled once per year. Abundances of monk parakeets and three native bird species (the Eurasian tree sparrow, the house sparrow, and the blackbird *Turdus merula*) were estimated by performing a census of these species in each park. The census was done at a walking speed of approximately 1 km/h, recording all monk parakeets, sparrows, and blackbirds, as well as monk parakeet nests and the number of nesting chambers within them, along a 25‐m strip on each side of the transect line. Surveys took place between 08:00 and 11:00 h and were conducted by either a single observer or two observers walking together, in which case data recording was agreed upon beforehand. To avoid duplicate counts, birds and nests were recorded only when the observer reached their location, except for flying birds within the observation range, which were recorded immediately upon detection. Monk parakeets and sparrows were surveyed in all 3 years (2021, 2022, and 2023). Sparrow counts in 2021 and 2022 did not distinguish between the two *Passer* species present in parks, but this distinction was made in 2023. Blackbirds were surveyed only in 2021 and 2022. We recorded both visual encounters of parakeets and the number of nest chambers to assess local abundance.

### Observational study 2: Use of parakeet nests by native species

Fieldwork was conducted during spring 2023, from mid‐April to mid‐May, coinciding with the peak breeding activity of both monk parakeets and most native bird species in Madrid (Senar et al., 2019). Observations were carried out in 252 monk parakeet nests located in 13 parks with high nest densities, with all nests within the selected areas being surveyed (Figure 1C). Nest observations were conducted between 08:00 and 11:00 h, the period of peak daily bird activity. Upon locating a tree with at least one monk parakeet nest, observers identified the tree species, counted the number of nests per tree, and recorded the number of nesting chambers per nest. Observations of 25 min of duration were done at a distance of approximately 20 m from the tree base using binoculars (252 nests × 25 min = 105 h of observation). During this period, the following data were recorded: (i) nest activity, defined by the presence of at least one monk parakeet pair occupying a chamber; (ii) native bird species visiting the nest; (iii) breeding behavior of native species, distinguishing between individuals likely to be nesting (confirmed breeding species) and those for which nesting activity was uncertain (potential breeding species). A native bird pair was classified as a “confirmed breeder” if at least one individual was observed (1) bringing nesting material, feeding chicks, or repeatedly entering and exiting the same location of the nest, or (2) chicks were visible in the nest. A native bird was classified as a “potential breeder” when individuals spent a significant amount of time at the nest but did not perform any of the behaviors described above. Immediately following the observations, 95 monk parakeet nests were inspected using a crane. These inspections allowed us to validate the accuracy of our behavioral classifications: Nestlings were found in over 90% of the chambers previously identified through binocular observations as being used by native breeding species.

### Data analysis

We analyzed our data building generalized linear mixed models (GLMMs) and applying an information‐theoretic framework with multimodel inference (Burnham & Anderson, 2002). We first identified the optimal random‐effects structure by fitting the full fixed‐effects model under a set of biologically plausible random terms and selecting the structure with the lowest Akaike information criterion corrected for small sample size (AIC_c_). Once the optimal random‐effects structure was selected, we ranked the a priori candidate fixed‐effects models by AIC_c_. To account for model uncertainty, inference was based on the confidence set of models accumulating ≥90% of Akaike weights (*w*_i_). We obtained parameter estimates, their 95% CIs, and predictions via full (unconditional) model averaging over this set. We considered that a predictor variable actually had an effect on the response variable only if its 95% CI did not span zero after model averaging.

The first set of models was fitted to analyze aggressive and intimidating interactions between monk parakeets and four native species. The dataset included all time intervals in which both interacting species were present, regardless of whether an aggression or intimidation event occurred. We modeled event rates as counts standardized by the number of potential targets, which was included as a log‐transformed offset. Aggression models assumed a negative binomial error distribution (log link), while intimidation models used a binomial distribution (logit link). For each species pair, we first evaluated random‐effects structures considering park, sampling interval, bait subplot, and their nested combinations. After selecting the optimal random‐effects structure, we defined a set of candidate fixed‐effects models including the interaction direction (initiator vs. receiver) and the number of potential aggressors/intimidators (testing null, single, additive, and interaction terms).

The second set of models aimed to assess relationships between monk parakeet density and sparrow and blackbird abundance. Density of birds was calculated using the Kilometric Abundance Index (KAI). Although traditionally defined as the number of individuals detected per kilometer (Preatoni et al., 2012), we adapted the index for monk parakeets by using nesting chambers as the detection unit—an approach commonly used in monk parakeet surveys (Molina et al., 2016). We prioritized chamber counts as a proxy for local parakeet abundance to minimize census bias: Unlike mobile flocks, whose detectability varies due to movement and behavior, nest structures are stationary and readily detectable. Furthermore, our data confirmed a positive correlation between chamber density and visual parakeet counts (Pearson's *r* = 0.55; *p* < 0.001), validating chamber density as a robust metric of colony size. The KAI was calculated using the following equation:

$$\mathrm{KAI}=\frac{N}{D\times T}$$

where *N* represents the number of individuals (or nest chambers) recorded per transect, *D* is the transect length (in kilometers), and *T* is the total sampling time (in hours).

We modeled the KAI of sparrows and blackbirds—rounded to the nearest integer to satisfy discrete count assumptions—using a negative binomial error distribution with a log link. As predictors, we included year and the KAI of monk parakeet nest chambers (adjusted for varying occupancy rates due to population control; Appendix S2), testing their single, additive, and interactive effects. Random‐effects structures considered were none, random intercepts for park or transect, and transects nested within parks.

Finally, the third set of models examined the abundance and species richness of native tenant species breeding in monk parakeet nests, as well as the factors influencing their occurrence. For these analyses, we used GLMMs with a negative binomial error distribution and a log link. Response variables included the total number of native bird pairs, native species richness, and the specific abundance of tree sparrows, stock doves, and rock pigeons. As predictors, we included the number of nest chambers and the number of occupying monk parakeet pairs, testing their single, additive, and interactive effects. Park was included as a random intercept. Analyses were conducted on the combined set of confirmed and potential native breeders, as results were consistent with those restricted to confirmed pairs (Appendix S3: Table S1).

All statistical analyses were conducted in R (R Core Team, 2022). We used the *glmmTMB* package (Brooks et al., 2017) for GLMMs, *MuMIn* (Bartoń, 2025) for multimodel inference and model averaging, *performance* (Lüdecke et al., 2021) to check for multicollinearity, and *DHARMa* (Hartig, 2022) for residual diagnostics. Model fit was summarized using marginal and conditional *R* ^2^ (Nakagawa & Schielzeth, 2013).

## RESULTS

### Foraging experiment: Agonistic interactions between monk parakeets and native birds

**FIGURE 2:**
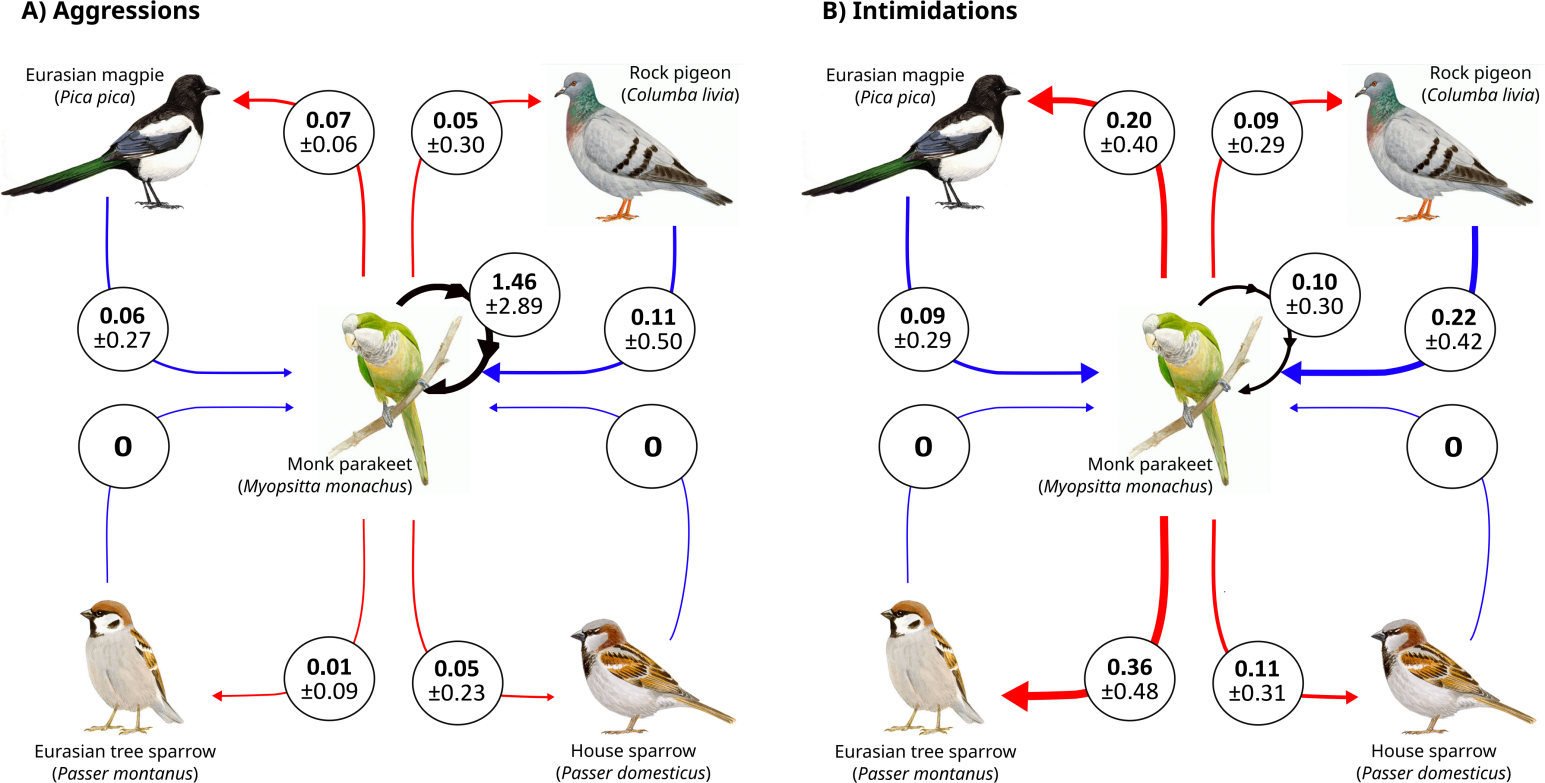
Frequency of aggressive (A) and intimidating (B) interactions between species pairs. Intraspecific aggression and intimidation frequencies are also shown for monk parakeets. Bold numbers represent mean values; values following the ± symbol indicate SDs. Arrow color indicates interaction directionality (red: from parakeet to native species; blue: from native species to parakeet; black: between parakeets). Arrow thickness increases with the mean frequency of interactions. Note that aggression and intimidation frequencies are not directly comparable, as aggression was recorded as the total number of events per interval, whereas intimidation was recorded only as a binary presence/absence variable per interval. All bird illustrations in this figure were created by Juan Varela Simó.

Each observer conducted 60 h of simultaneous observations, totaling 120 h. During this period, and based on individual observation intervals, a total of 4622 monk parakeets, 2855 rock pigeons, 2392 Eurasian magpies, 627 Eurasian tree sparrows, and 212 house sparrows were recorded. The number of aggressive events and the presence or absence of intimidation were measured for each interacting species pair (Appendix S4: Figure S1). While aggressive interactions involving monk parakeets were predominantly intraspecific—occurring mainly among themselves—intimidations were mostly interspecific. The most distinct patterns observed were parakeets intimidating tree sparrows and magpies, and, conversely, rock pigeons intimidating parakeets (Figure 2).

**FIGURE 3:**
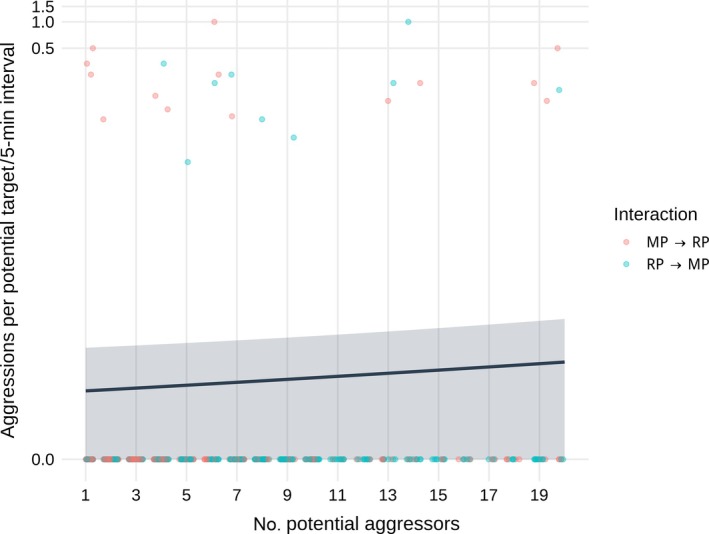
Model‐averaged predictions of aggression rate between monk parakeets and rock pigeons as a function of the number of potential aggressors. The solid dark line represents the overall predicted aggression rate (number of aggressions per potential target per 5‐min interval), with the shaded band indicating the 95% CI. Because interaction directionality did not have a statistically significant effect (its 95% CI spanned zero), a single global trend is shown. However, observed raw rates (points) are colored to display the underlying data distribution for each interaction direction (red: monk parakeet → rock pigeon; blue: rock pigeon → monk parakeet). MP, monk parakeet; RP, rock pigeon.

Regarding interspecific aggressions, model‐averaged coefficients (Appendix S5: Table S1) revealed that only the number of potential aggressors had a robust effect for the monk parakeet–rock pigeon interaction (95% CI: [0.03, 0.15]). Specifically, the overall frequency of aggression increased with bird density, regardless of which species initiated the attack (Figure 3). For interactions involving magpies, none of the variables evaluated showed a robust effect on aggression rates, as all model‐averaged 95% CIs spanned zero. Finally, for interactions involving tree sparrows and house sparrows, the extremely low frequency of aggressive events resulted in models with high uncertainty and massive SEs (Appendix S5: Table S1), preventing the identification of any robust predictors for these two species.

**FIGURE 4:**
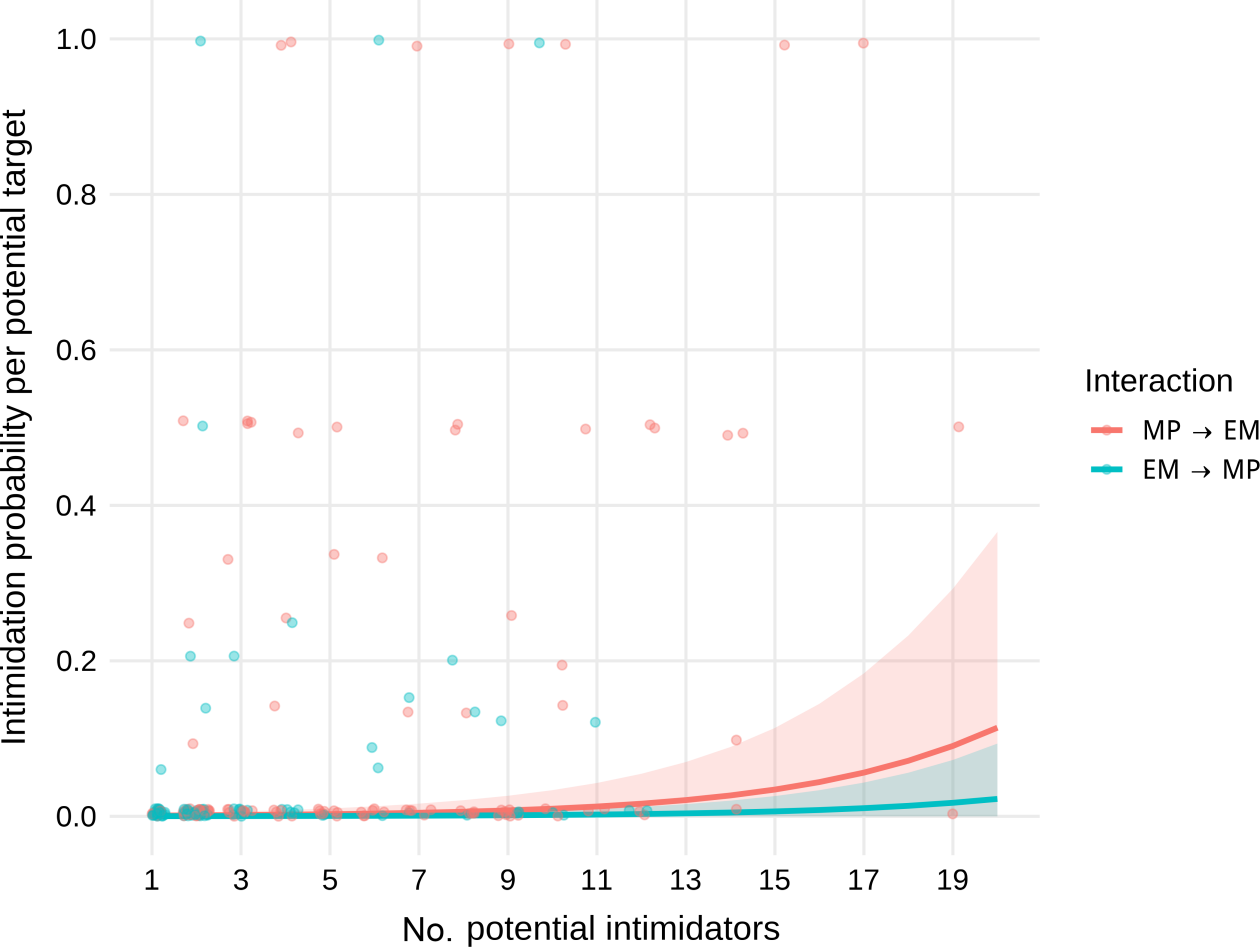
Model‐averaged predictions of intimidation probability between monk parakeets and Eurasian magpies as a function of the number of potential intimidators. Solid lines represent the predicted probability of intimidation per potential target, and shaded bands indicate 95% CIs. Points show observed raw rates (jittered for visibility). Colors denote interaction direction (red: monk parakeet → Eurasian magpie; blue: Eurasian magpie → monk parakeet). Both species show an increase in intimidation probability with bird density, with monk parakeets remaining the dominant intimidators across the observed range. While the interaction term was not statistically robust (95% CI spanned zero), full model averaging includes its mathematical weight, resulting in the slight divergence of the trends at higher densities. Notably, field observations for magpies intimidating parakeets (blue points) were restricted to groups of up to 11 individuals; therefore, predictions beyond this density represent model extrapolations. EM, Eurasian magpie; MP, monk parakeet.

In the case of interspecific intimidations, model‐averaged estimates indicated that no variables had a robust effect on the probability of monk parakeet–rock pigeon intimidations, as all 95% CIs spanned zero (Appendix S5: Table S2). In contrast, for interactions involving Eurasian magpies, monk parakeets were the dominant intimidators (directionality 95% CI: [0.38, 1.48]), and the overall probability of intimidation rose with the number of potential intimidators (95% CI: [0.06, 0.26]; Figure 4). The lack of a robust interaction effect indicates both species increased intimidation rates similarly as bird density rose. Notably, predictions at high densities remain highly uncertain, as no magpie‐initiated intimidations were recorded in groups exceeding 11 individuals. Finally, similar to the aggression results, the extremely low frequency of intimidation events involving sparrows resulted in models with high uncertainty and massive SEs (Appendix S5: Table S2), preventing the identification of any robust predictors.

### Observational study 1: Correlations between monk parakeet and native bird abundances

**FIGURE 5:**
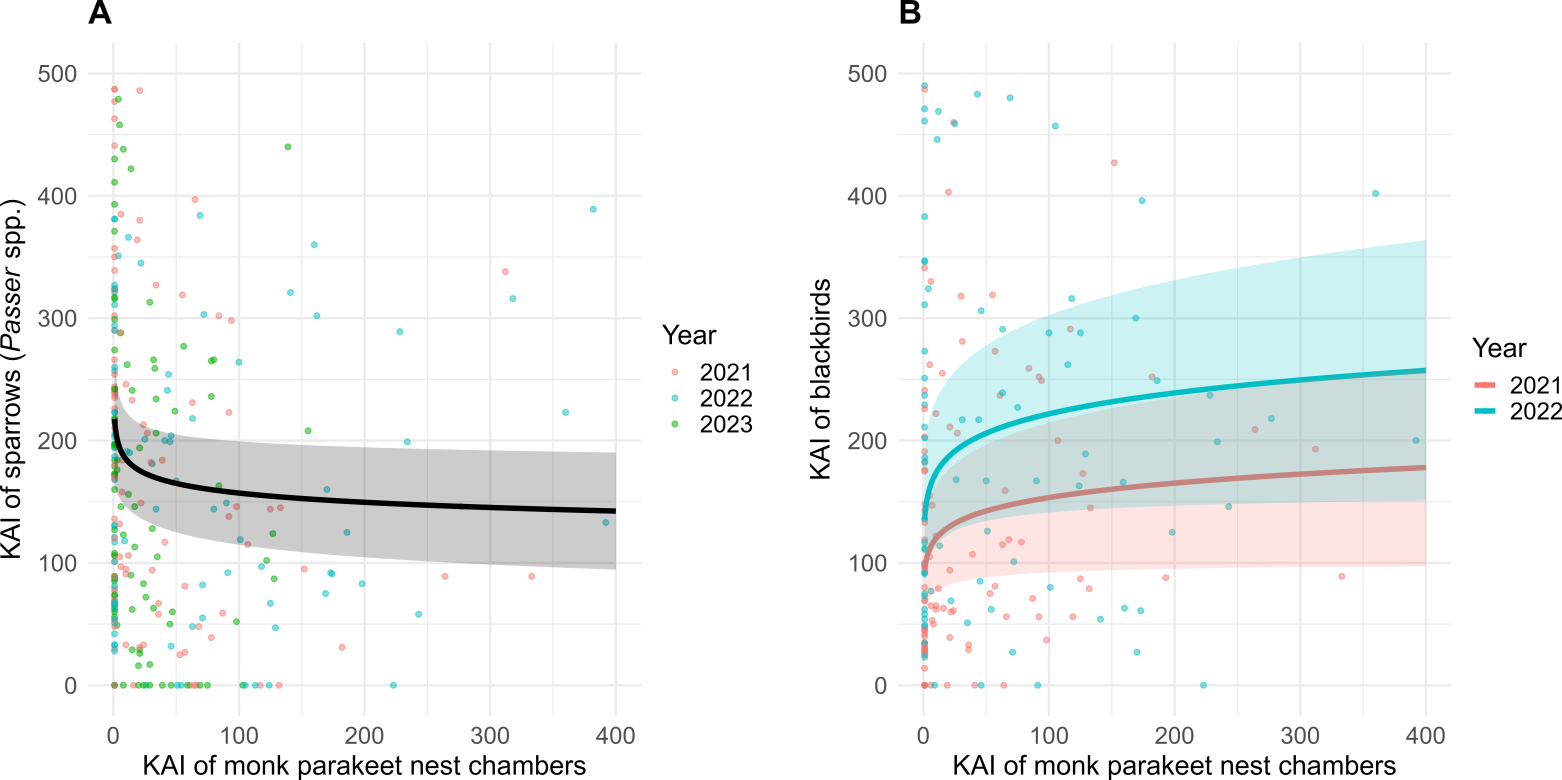
Relationships between the Kilometric Abundance Index (KAI) of monk parakeet nest chambers and the KAI of (A) sparrows (*Passer* spp.) and (B) blackbirds. Sparrows were recorded in 2021, 2022, and 2023, whereas blackbirds were recorded in 2021 and 2022. Lines show model‐averaged generalized linear mixed model (GLMM) predictions on the original KAI scale, with shaded areas representing 95% CIs. In panel (A), a single overall trend line (black) is shown because the effect of year was not statistically significant, whereas in Panel (B), year‐specific trend lines (colored) are displayed. Points represent observed KAI values for individual transects, colored by sampling year.

Sparrow (*Passer* spp.) abundance (KAI) was negatively associated with the density of monk parakeet nest chambers (Figure 5A; Appendix S6: Table S1). In contrast, blackbird abundance showed a positive association with nest chamber density, alongside a significant effect of sampling year (Figure 5B; Appendix S6: Table S1).

Additionally, to assess whether pooling the *Passer* genus masked species‐specific responses, we evaluated the 2023 data for tree sparrows and house sparrows both separately and jointly. In all three cases, we found no robust association between nest chamber density and sparrow abundance, as all model‐averaged 95% CIs spanned zero (Appendix S6: Table S1).

### Observational study 2: Use of parakeet nests by native species

Among the 252 monk parakeet nests surveyed, 130 (52%) were actively occupied by at least one monk parakeet pair, hosting a total of 151 parakeet pairs. We recorded 11 native bird species using these structures, comprising 191 actively nesting pairs and 40 potentially nesting pairs (Appendix S7: Figure S1). Assuming all potential pairs bred, 48% of the nests (*N* = 252) hosted at least one native pair, with 20% containing multiple pairs. In terms of species richness, 35% of the nests hosted a single native species, 12% contained two, and 1% (three nests) contained three. The most abundant native tenants were the tree sparrow (48% of recorded pairs), stock dove (38%), and rock pigeon (7%) (Appendix S7: Figure S1).

**FIGURE 6:**
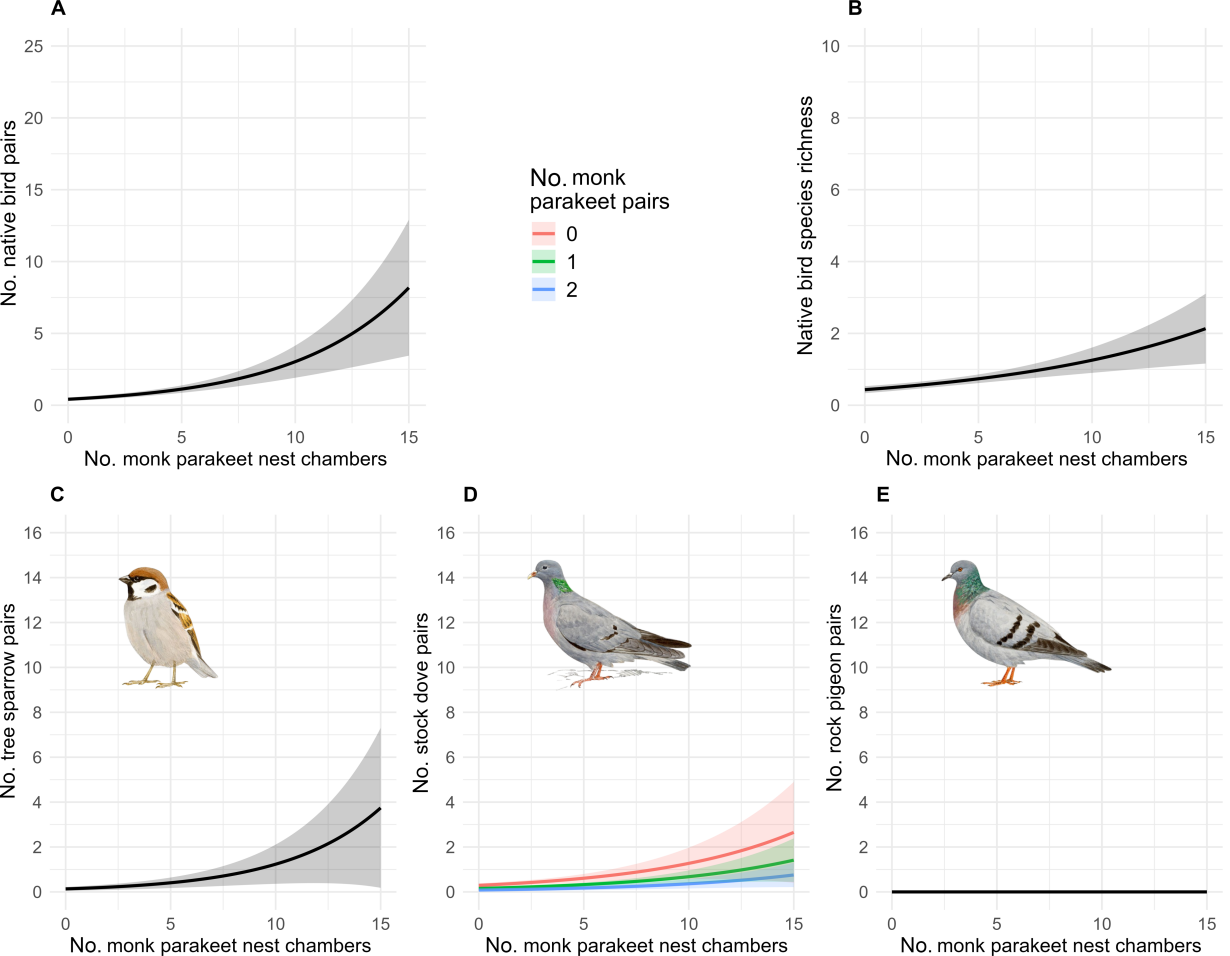
Model‐averaged predictions of the number of native birds breeding in monk parakeet nests as a function of the number of monk parakeet nest chambers and the number of monk parakeet pairs occupying them. Panels show (A) total number of native bird pairs, (B) species richness of native birds, (C) number of tree sparrow pairs, (D) number of stock dove pairs, and (E) number of rock pigeon pairs. For variables where the presence of occupying monk parakeets had no significant effect (Panels A, B, C, and E), a single overall trend line (black) is displayed based solely on the number of nest chambers. For stock doves (Panel D), lines represent predicted values for different numbers of monk parakeet pairs (colors), showing parallel trends that reflect additive effects without interaction. Shaded areas represent 95% CIs; axes are shown on the original scales. Predictions at the highest values of nest chambers and parakeet pairs are based on few observations and should be interpreted with caution. In Panel (E), although Appendix S3: Table S1 reports a statistically significant positive coefficient, the predicted absolute abundance for the rock pigeon remains a fraction of a pair due to its lower frequency in our sample. When plotted on this shared 0–16 scale, this visually presents as a flat line. All bird illustrations in this figure were created by Juan Varela Simó.

Model‐averaged estimates revealed that the total number of native breeding pairs, native species richness, and the specific abundances of tree sparrows and rock pigeons were positively associated solely with the number of parakeet nest chambers (Figure 6A–C,E; Appendix S3: Table S1). For these metrics, the number of occupying monk parakeet pairs had no robust effect, as their 95% CIs spanned zero. In contrast, stock dove abundance was positively associated with the number of nest chambers but negatively associated with the number of occupying monk parakeets, reflecting additive, noninteractive effects of both variables (Figure 6D; Appendix S3: Table S1).

## DISCUSSION

Although humans have repeatedly introduced bird species into novel environments, their effects on native species remain poorly understood (Evans & Blackburn, 2020). By evaluating both competitive and commensal interactions, our study provides new insights into the complex web of relationships between invasive monk parakeets and native birds in urban areas.

To evaluate our first hypothesis—that dominance during competition for shared food resources is determined by body size—we quantified agonistic interactions (aggressions and intimidations) during a foraging experiment. Contrary to our expectations, our results did not support a strict size‐mediated dominance hierarchy. We predicted that the medium‐sized monk parakeet would exert physical dominance over smaller native birds; however, physical aggressions involving the smallest species in our assemblage—both sparrow taxa—were virtually absent. Conversely, we expected parakeets to be subordinate to larger species; yet, physical aggressions with the largest species, the rock pigeon, were completely symmetrical, simply escalating for both species as competitor density increased. Similarly, physical encounters with Eurasian magpies showed no clear dominance by either species. Intimidation dynamics further contradicted the size‐based dominance prediction. As with physical conflicts, intimidations involving sparrows or rock pigeons were negligible or lacked clear directional patterns. In contrast, parakeets clearly dominated intimidations against the Eurasian magpie, even though the overall frequency of these encounters was low. Although the probability of intimidation increased for both species as bird density grew, parakeets consistently maintained their baseline dominance. Altogether, these results align with previous studies reporting tolerant foraging behavior in monk parakeets when interacting with species sharing the same feeding sites (Briceño et al., 2019; Di Santo et al., 2016).

The scarcity of direct agonistic interactions involving sparrows should not necessarily be interpreted as an absence of general competition. The encounters recorded here represent only a subset of the full competitive dynamics at play, as our experimental design was restricted to species simultaneously present at the ground feeding stations. The virtual absence of direct conflict at ground level may actually reflect size‐mediated passive dominance, where larger species displace smaller ones to alternative feeding sites or strata without active physical aggression or intimidation (Alatalo et al., 1987; Francis et al., 2018), precluding the direct observation of these competitive events. Indeed, during our field trials, tree sparrows frequently remained on the periphery of the feeding sites rather than mixing with larger species. This observation aligns with the cautious foraging behaviors typically exhibited by this species, reflecting a natural tendency to avoid dominant individuals or to stay close to protective cover (Liker & Barta, 2002). The presence of feeding flocks of monk parakeets may exacerbate this cautious behavior, effectively translating into spatial displacement and restricted access to the central food resource. Remarkably similar patterns of spatial segregation have been documented for invasive rose‐ringed parakeets (*Psittacula krameri*), whose presence forced smaller native species to the periphery of feeding sites (Le Louarn et al., 2016) and significantly reduced their visitation rates at urban feeders (Peck et al., 2014).

To evaluate our second hypothesis—that spatial displacement and abundance variations are driven by the degree of ecological similarity—we analyzed the relationship between parakeet density and native bird abundances across urban parks. While directly linking individual behavioral avoidance to demographic consequences requires long‐term survival and reproductive data (Baker et al., 2014; Dhondt, 2012), broad‐scale abundance correlations provide a crucial first step to detect population‐level impacts. We predicted that increasing parakeet density would be negatively associated with the abundance of smaller species sharing high dietary overlap, such as sparrows. Although correlational, our models indicate that increasing parakeet nest chamber density is associated with lower sparrow (*Passer* spp.) abundance. This result supports our prediction that high niche overlap increases vulnerability to negative impacts. In this scenario, local interference competition may force sparrows into suboptimal habitats, ultimately lowering their local population densities. This pattern contrasts with previous reports of peaceful cohabitation (Batllori & Nos, 1985; Briceño et al., 2019; Hernández‐Brito et al., 2021) and contradicts positive associations found in comparable urban studies (Appelt et al., 2016). Our findings are of particular concern given the widespread decline of sparrows across Europe (Burns et al., 2021; Šálek et al., 2015; Staneva & Burfield, 2017). To ensure that pooling species did not mask conflicting species‐specific trends, we analyzed the 2023 dataset where both taxa were distinguished. While this single‐year analysis yielded nonsignificant results for both species, it confirmed the absence of diametrically opposed responses. Importantly, the lack of significance in 2023 is consistent with the intensive parakeet control program, which severely compressed the upper end of the parakeet density gradient in that year, thereby limiting the statistical power to detect spatial avoidance. This validates our aggregate approach, demonstrating that integrating data from all 3 years is essential to encompass the full invasion gradient and provide the statistical power necessary to detect the underlying negative spatial association. Crucially, although we lack direct demographic data, ecological theory posits that chronic displacement from optimal foraging sites can reduce fitness by limiting energy intake and increasing stress (Creel et al., 2007; Lima & Dill, 1990). This mechanism is consistent with findings by Peck et al. (2014), who demonstrated that the mere presence of invasive parakeets significantly reduces the visitation rates of native birds to feeding resources. Such cumulative exclusion provides a plausible mechanistic explanation for the lower sparrow abundances observed in highly invaded areas.

In contrast to the negative association observed for sparrows, common blackbird abundance showed a significant positive correlation with parakeet density. This outcome aligns with our second hypothesis, as the blackbird—a species of similar size to the monk parakeet but with divergent dietary requirements—was expected to exhibit a response distinct from the competitive displacement predicted for smaller species with higher niche overlap. This finding contradicts previous anecdotal claims that monk parakeets might displace blackbirds through food competition (Batllori & Nos, 1985), aligning instead with studies reporting neutral or positive associations with other ecologically similar thrushes (e.g., *Turdus migratorius*; Appelt et al., 2016). The underlying mechanism is likely driven by shared habitat preferences (i.e., habitat filtering; Shochat et al., 2010) where both species select similar urban park features, alongside a partial dietary segregation that minimizes direct interference (granivorous‐frugivorous parakeets vs. omnivorous‐insectivorous blackbirds; Snow, 1958; South & Pruett‐Jones, 2000). Alternatively, a mutually compatible hypothesis involves heterospecific attraction: The conspicuous vocal activity and colonial nature of monk parakeets may function as a social cue for blackbirds, signaling a productive habitat or providing “sentinel” benefits through social information on predation risk (Mönkkönen et al., 1990; Thomson et al., 2006).

Finally, our results support the role of monk parakeets as ecosystem engineers, providing nesting opportunities for native birds, particularly tree sparrows and stock doves, which together accounted for 86% of all recorded native breeding pairs. This pattern contrasts with Hernández‐Brito et al. (2021), who reported house sparrows as the most frequent tenants. This discrepancy likely reflects regional differences, as their study encompassed both urban and rural populations in southern Spain. When examining the drivers of this use, our models consistently showed that the overall abundance of native breeding pairs, native species richness, and the specific abundances of tree sparrows, stock doves, and rock pigeons all increased significantly with the number of nest chambers. This strongly supports our third hypothesis, confirming that the physical size of the nest structure drives commensal nest use. However, the influence of occupying parakeets varied among the native tenants. Specifically, stock dove abundance declined significantly as the number of parakeet pairs increased, confirming that their nest use is largely limited to vacant chambers free from parakeet interference (Briceño et al., 2019). In contrast, parakeet presence had no significant effect on the abundance of rock pigeons, tree sparrows, overall native bird abundance, or species richness. For tree sparrows, this lack of competitive exclusion likely reflects their ability to use the external nest structure as a substrate for building their own nests rather than competing for internal chambers (Hernández‐Brito et al., 2021), allowing them to co‐nest successfully regardless of parakeet activity. Consequently, the value of these structures for native avifauna is primarily driven by their size (number of chambers) rather than their occupancy status. While large, abandoned nests are essential for species sensitive to interference like the stock dove, active parakeet colonies remain highly valuable breeding hotspots for more tolerant species like the tree sparrow. These findings have urgent management implications. Since parakeet nests function as breeding hotspots for native avifauna, their indiscriminate mechanical removal during the breeding season poses a significant risk to native species. This caution applies to all large nests, which harbor the highest native biodiversity, regardless of whether they are actively used by parakeets or abandoned. Management protocols must therefore prioritize the timing of interventions to avoid destroying active native broods, balancing invasive species control with the conservation of nontarget native species.

Despite these nesting opportunities, this use by native species must be viewed with caution, as monk parakeet nests may involve a trade‐off between structural benefits and increased sanitary risks. The high host density and accumulation of organic material in perennial nests, combined with a stable microclimate, can foster high parasite loads, potentially turning these structures into ecological traps for native birds (Aramburú et al., 2003; Caccamise & Weathers, 1977). Moreover, close interaction with invasive parakeets raises concerns about pathogen spillover and the amplification of novel diseases within the recipient community (Aramburú et al., 2003; Blanco‐González et al., 2024; López et al., 2023; Mori et al., 2019). Thus, the conservation value of these nests is conditional on their sanitary status. Future management strategies should not only regulate nest removal but also incorporate epidemiological surveillance to ensure that preserving these structures does not inadvertently compromise the health of the native avian community.

## LIMITATIONS AND FURTHER PROSPECTS

While our study provides valuable insights into the ecological interactions between monk parakeets and native avifauna, several limitations warrant consideration to guide future research. First, our data on individual behavior and nest occupancy cannot be directly extrapolated to population demographics. We lack evidence on whether these interactions affect native species' abundance or recruitment rates, preventing definitive conclusions about long‐term population growth or decline. Second, regarding sampling heterogeneity, the taxonomic resolution of *Passer* species varied across years (pooled in the first 2 years vs. distinguished in 2023), and blackbird counts were limited to 2 years. While this prevents a detailed year‐to‐year comparison at the species level, our aggregate approach captures general interaction trends that require extended standardized monitoring to be fully confirmed. Finally, high nest occupancy does not necessarily imply high habitat quality. Lacking data on reproductive success and pathogen prevalence, we cannot yet determine whether these structures serve as beneficial shelters or “ecological traps” where native fitness is compromised by disease or interspecific harassment.

## CONCLUSIONS

These findings confirm that the ecological role of invasive monk parakeets cannot be categorized simply as “positive” or “negative,” but rather as a dual dynamic that creates a significant management dilemma. On the one hand, parakeets exert some competitive pressure during foraging—primarily affecting abundant, non‐threatened species—and show negative spatial correlations with declining urban sparrows that warrant further attention. On the other hand, they simultaneously act as ecosystem engineers by providing large nesting structures that are highly valuable to native biodiversity. Consequently, management strategies relying on indiscriminate nest removal may inadvertently harm native populations by destroying essential breeding sites. However, current evidence cannot guarantee the long‐term safety of this arrangement, primarily due to unquantified trade‐offs such as pathogen transmission within shared nests. We argue that management must therefore evolve from simple eradication tactics toward evidence‐based, holistic approaches. Such strategies should carefully weigh the loss of nesting resources against the potential sanitary costs, as well as the broader impacts on human infrastructure and agriculture (Reed et al., 2014; Senar et al., 2016), when deciding the fate of these invasive populations.

## AUTHOR CONTRIBUTIONS

Jon Blanco‐González, Isabel López‐Rull, Fernando Enríquez, and Luis Cayuela conceived the ideas and designed the methodology. Jon Blanco‐González collected the data. Jon Blanco‐González and Luis Cayuela analyzed the data. Jon Blanco‐González, Isabel López‐Rull, and Luis Cayuela wrote the manuscript. All authors contributed critically to the drafts and gave final approval for publication.

## CONFLICT OF INTEREST STATEMENT

Co‐authors Jon Blanco‐González and Fernando Enríquez are employees contracted by MATINSA, the company awarded the contract by the Madrid City Council to control the monk parakeet population in Madrid from May 2021 to April 2023.

## Supporting information


Appendix S1:

Appendix S2:

Appendix S3:

Appendix S4:

Appendix S5:

Appendix S6:

Appendix S7:

## Data Availability

Data and code (Blanco‐González et al., 2026) are available from e‐cienciaDatos at https://doi.org/10.21950/1IVRNO.
