# Supplementary material for: Balancing conflict and coexistence: Interactions between invasive monk parakeets and native urban birds
Source: Ecol Appl. 2026 Jun 18;36(4):e70275. doi: 10.1002/eap.70275 (PMC13276877; doi:10.1002/eap.70275)
Supplement: Supplementary file 1 — Appendix S1: [file EAP-36-e70275-s003.pdf]

Appendix S1

Balancing conflict and coexistence: Interactions between invasive monk parakeets and native urban birds

Jon Blanco-González, Isabel López-Rull, Fernando Enríquez and Luis Cayuela

Ecological Applications

Appendix S1: Spatial configuration of the experimental feeders

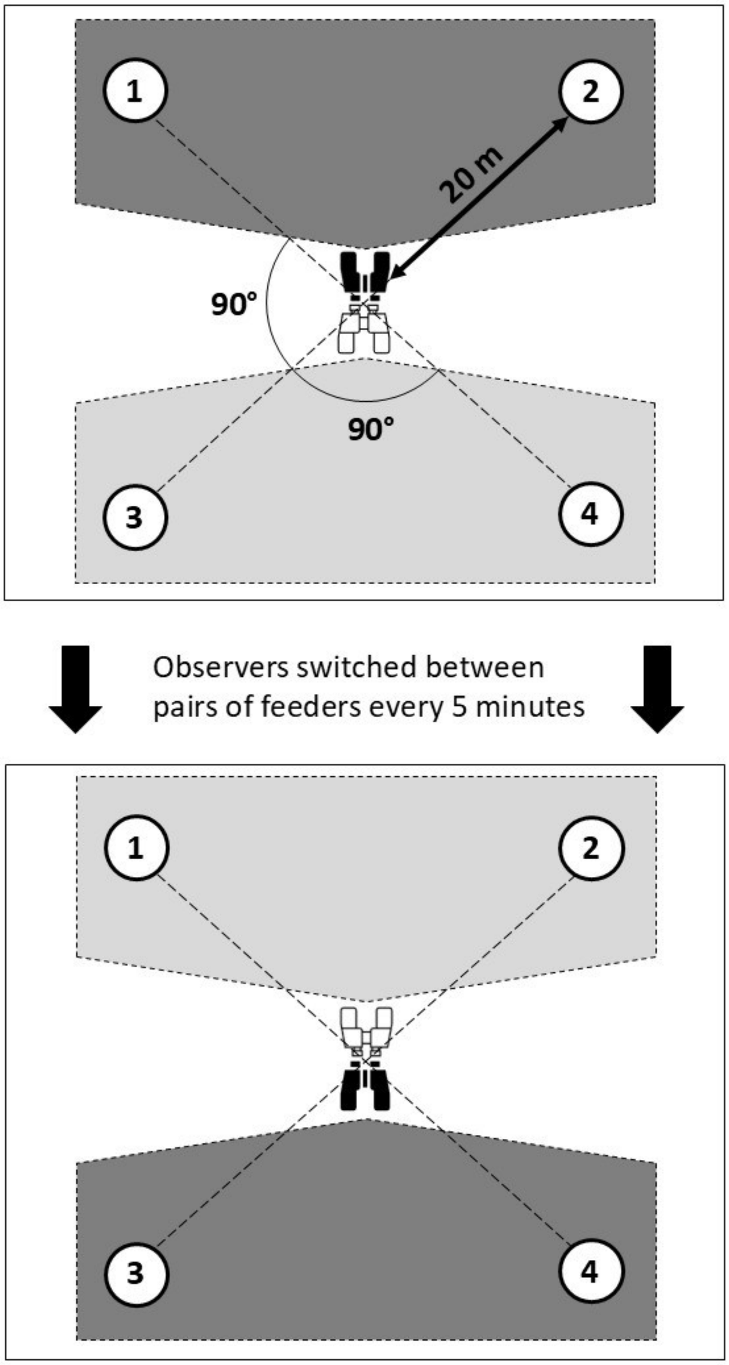

Figure S1. Diagram illustrating the positions of the two observers relative to the feeders, which are labelled with numbers from 1 to 4. Each feeder was placed approximately 20 m from the observers. Observer 1 is depicted with black binoculars and the feeders they observed are shown in dark gray, while observer 2 is depicted with white binoculars and the feeders they observed are shown in pale gray. The figure was created by Jon Blanco-González using binocular icons from Microsoft PowerPoint.
