# Supplementary material for: Balancing conflict and coexistence: Interactions between invasive monk parakeets and native urban birds
Source: Ecol Appl. 2026 Jun 18;36(4):e70275. doi: 10.1002/eap.70275 (PMC13276877; doi:10.1002/eap.70275)
Supplement: Supplementary file 2 — Appendix S2: [file EAP-36-e70275-s002.pdf]

Appendix S2

Balancing conflict and coexistence: Interactions between invasive monk parakeets and native urban birds

Jon Blanco-González, Isabel López-Rull, Fernando Enríquez and Luis Cayuela

Ecological Applications

Appendix S2: Adjustment of parakeet abundance proxy (effective nest chambers)

Madrid implemented an intensive monk parakeet control program between May 2021 and April 2023 (Blanco-González et al., 2025), necessitating the recalculation of nest chamber occupancy rates for 2022 and 2023 to estimate the parakeet population in the study parks. Based on a pre-management occupancy of 1.2–1.39 parakeets per chamber (Nebreda et al., 2019) and estimated parakeet numbers in 2022 and 2023 (Blanco-González et al., 2025), we assumed that chamber occupancy declined proportionally to the total population reduction. Using this approach, occupancy was estimated as:

$$Occupancy \in 2022 = Occupancy \in 2021 \times \left(\frac{Parakeetpopulation \in 2022}{Parakeetpopulation \in 2021}\right)$$

$$Occupancy \in 2023 = Occupancy \in 2021 \times \left(\frac{Parakeetpopulation \in 2023}{Parakeetpopulation \in 2021}\right)$$

This yielded occupancy estimates of 1.12 parakeets per chamber in 2022 and 0.76 in 2023.

However, since we aimed to use the number of nest chambers rather than parakeet numbers as an explanatory variable, a correction factor was applied to adjust the number of occupied chambers in 2022 and 2023 based on changes in average occupancy per chamber. The correction factor was calculated as the ratio between the mean occupancy in a given year and the mean occupancy in 2021:

$$Correctionfactorfor2022 = \frac{1.12}{1.295} = 0.865$$

$$Correctionfactorfor2023 = \frac{0.76}{1.295} = 0.587$$

Thus, to estimate the effective number of occupied chambers in 2022 and 2023, the total recorded chambers in each park were multiplied by 0.865 and 0.587, respectively.

References

Blanco-González, J., L. Cayuela, F. Enríquez, and I. López-Rull. 2025. Developing best management practices for the invasive Monk Parakeet (Myiopsitta monachus) in urban environments. NeoBiota 98:163–196. https://doi.org/10.3897/neobiota.98.132982

Nebreda, A., E. Escudero, and J. del Moral. 2019. Censo de Cotorra Argentina en el municipio de Madrid. SEO/BirdLife, Madrid.
