# Supplementary material for: Balancing conflict and coexistence: Interactions between invasive monk parakeets and native urban birds
Source: Ecol Appl. 2026 Jun 18;36(4):e70275. doi: 10.1002/eap.70275 (PMC13276877; doi:10.1002/eap.70275)
Supplement: Supplementary file 3 — Appendix S3: [file EAP-36-e70275-s001.pdf]

Appendix S3

Balancing conflict and coexistence: Interactions between invasive monk parakeets and native urban birds

Jon Blanco-González, Isabel López-Rull, Fernando Enríquez and Luis Cayuela

Ecological Applications

Appendix S3: Model selection results for native nest occupancy

This appendix details the multimodel inference results for the overall abundance and species richness of native birds, as well as the specific abundance of tree sparrows), stock doves, and rock pigeons nesting in monk parakeet nests (**Table S1**).

To assess the robustness of our findings, model selection was conducted separately for two datasets. The first dataset ("All records") included both confirmed breeding pairs and probable nesting attempts. The second dataset ("Confirmed breeders") was restricted exclusively to pairs with confirmed breeding evidence.

The table below displays the full selection process in two sections:

1. **Random effects structure:** Comparison of alternative random-effects structures based on the lowest AICc.
2. **Fixed effects selection:** Ranking of candidate models (fixed-effects models) using the selected random structure. Inference is based on the confidence set of models accumulating ≥ 90% of Akaike weights (w_i).

The tables report parameter estimates (β ± SE) and their 95% confidence intervals [95% CI] derived from unconditional model averaging over the confidence set. Marginal (R^2m) and conditional (R^2c) coefficients of determination are reported for the top-ranked model in each analysis.

Table S1. Model selection and model-averaged coefficients for the abundance and richness of native bird pairs nesting in monk parakeet nests. Results are presented separately for all observed nesting attempts ("All observations") and for confirmed breeding pairs ("Just confirmed breeders"). As all records for the rock pigeon corresponded to confirmed breeding, results for this species are identical across both datasets.

	Abundance of native birds	Richness of native birds	Abundance of tree sparrows	Abundance of stock doves	Abundance of rock pigeon
**All observations**					
**Random effects structure**					
No random factors	593.91	**479.88**	384.30	**353.86**	82.50
Park	**593.68**	481.65	**373.36**	355.82	**50.98**

	**Fixed effects selection**					
	Null model	654.02 (0.00)	504.13 (0.00)	412.94 (0.00)	367.76 (0.00)	58.75 (0.01)
	N° of chambers	**593.22 (0.40)**	483.75 (0.07)	**375.24 (0.25)**	361.67 (0.01)	53.27 (0.08)
	N° of parakeets	650.70 (0.00)	506.20 (0.00)	405.65 (0.00)	366.41 (0.00)	60.17 (0.00)
	N° of chambers + n° of parakeets	**593.99 (0.28)**	**480.28 (0.42)**	**376.64 (0.12)**	**352.22 (0.69)**	**49.00 (0.67)**
	N° of chambers × n° of parakeets	**593.68 (0.32)**	**479.89 (0.51)**	**373.36 (0.63)**	**353.86 (0.30)**	**50.98 (0.25)**
	R^2m / R^2c	0.82 / 1.00	—	0.45 / 1.00	—	0.01 / 1.00
	**Model-averaged coefficients**					
	N° of chambers (β ± SE)	**0.22 ± 0.04 [0.14, 0.30]**	**0.16 ± 0.04 [0.08, 0.24]**	**0.27 ± 0.06 [0.15, 0.39]**	**0.15 ± 0.04 [0.07, 0.23]**	**0.52 ± 0.11 [0.30, 0.74]**
	N° of parakeets (β ± SE)	-0.01 ± 0.15 [-0.30, 0.28]	-0.17 ± 0.21 [-0.58, 0.24]	0.42 ± 0.36 [-0.29, 1.13]	**-0.59 ± 0.24 [-1.06, -0.12]**	-1.35 ± 0.90 [-3.11, 0.41]
	N° of chambers × N° of parakeets (β ± SE)	-0.01 ± 0.02 [-0.05, 0.03]	-0.02 ± 0.03 [-0.08, 0.04]	-0.05 ± 0.05 [-0.15, 0.05]	-0.01 ± 0.03 [-0.07, 0.05]	0.01 ± 0.07 [-0.13, 0.15]
**Just confirmed breeders**						
	**Random effects structure**					
	No random factors	**529.66**	**418.66**	347.48	**303.63**	82.50
	Park	531.63	420.75	**340.31**	305.38	**50.98**
	**Fixed effects selection**					
	Null model	591.86 (0.00)	446.61 (0.00)	376.26 (0.00)	321.05 (0.00)	58.75 (0.01)
	N° of chambers	**528.35 (0.51)**	422.21 (0.08)	**343.34 (0.16)**	311.72 (0.01)	53.27 (0.08)
	N° of parakeets	585.56 (0.00)	448.54 (0.00)	368.26 (0.00)	320.37 (0.00)	60.17 (0.00)
	N° of chambers + n° of parakeets	**530.02 (0.22)**	**418.84 (0.44)**	**343.70 (0.13)**	**301.99 (0.69)**	**49.00 (0.67)**
	N° of chambers × n° of parakeets	**529.66 (0.27)**	**418.66 (0.48)**	**340.31 (0.71)**	**303.63 (0.30)**	**50.98 (0.25)**
	R^2m / R^2c	—	—	0.47 / 1.00	—	0.01 / 1.00
	**Model-averaged coefficients**					
	N° of chambers (β ± SE)	**0.25 ± 0.04 [0.17, 0.33]**	**0.18 ± 0.04 [0.10, 0.26]**	**0.30 ± 0.07 [0.16, 0.44]**	**0.19 ± 0.04 [0.11, 0.27]**	**0.52 ± 0.12 [0.28, 0.76]**
	N° of parakeets (β ± SE)	0.03 ± 0.16 [-0.28, 0.34]	-0.20 ± 0.23 [-0.65, 0.25]	0.61 ± 0.41 [-0.19, 1.41]	**-0.67 ± 0.28 [-1.22, -0.12]**	-1.35 ± 0.92 [-3.15, 0.45]
	N° of chambers × N° of parakeets (β ± SE)	-0.01 ± 0.03 [-0.07, 0.05]	-0.02 ± 0.03 [-0.08, 0.04]	-0.06 ± 0.05 [-0.16, 0.04]	-0.01 ± 0.03 [-0.07, 0.05]	0.01 ± 0.07 [-0.13, 0.15]

Note: In the "Fixed effects selection" section, values in parentheses indicate Akaike weights (w^i). In the "Model-averaged coefficients" section, values in brackets indicate the 95% confidence intervals [95% CI]. Bold text denotes the selected random-effects structure, the models included in the 90% confidence set, and the 95% CIs that do not span zero. The symbol "—" indicates parameters that are either not applicable, not retained in the top model set, or where the null model was the top-ranked model (rendering R^2 values negligible or undefined).
