# Supplementary material for: Balancing conflict and coexistence: Interactions between invasive monk parakeets and native urban birds
Source: Ecol Appl. 2026 Jun 18;36(4):e70275. doi: 10.1002/eap.70275 (PMC13276877; doi:10.1002/eap.70275)
Supplement: Supplementary file 4 — Appendix S4: [file EAP-36-e70275-s005.pdf]

Appendix S4

Balancing conflict and coexistence: Interactions between invasive monk parakeets and native urban birds

Jon Blanco-González, Isabel López-Rull, Fernando Enríquez and Luis Cayuela

Ecological Applications

Appendix S4: Matrices of agonistic interactions

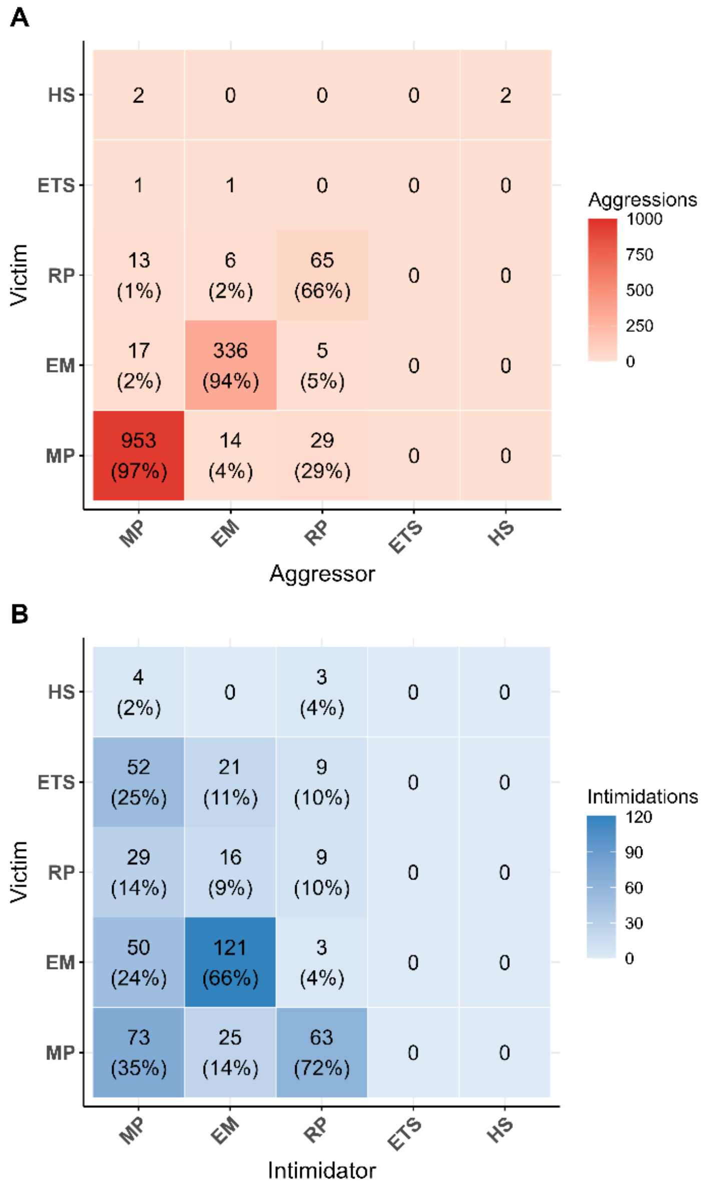

Figure S1. Number of aggressive (**A**) and intimidation (**B**) events among five bird species: monk parakeet (MP), Eurasian magpie (EM), rock pigeon (RP), Eurasian tree sparrow (ETS), and house sparrow (HS). The x-axis indicates the aggressor or intimidator species, while the y-axis represents the target species. Each cell shows the total number of recorded events, with percentages in parentheses indicating how the actions of each aggressor or intimidator were distributed across the different target species. Note that aggression and intimidation frequencies are not directly comparable, as aggression was recorded as the total number of events per interval, whereas intimidation was recorded only as a binary presence/absence variable per interval.
