# Supplementary material for: Balancing conflict and coexistence: Interactions between invasive monk parakeets and native urban birds
Source: Ecol Appl. 2026 Jun 18;36(4):e70275. doi: 10.1002/eap.70275 (PMC13276877; doi:10.1002/eap.70275)
Supplement: Supplementary file 5 — Appendix S5: [file EAP-36-e70275-s007.pdf]

Appendix S5

Balancing conflict and coexistence: Interactions between invasive monk parakeets and native urban birds

Jon Blanco-González, Isabel López-Rull, Fernando Enríquez and Luis Cayuela

Ecological Applications

Appendix S5: Model selection results for agonistic interactions

This appendix details the multimodel inference results for aggression (**Table S1**) and intimidation (**Table S2**) rates between monk parakeets (MP) and native species: rock pigeon (RP), Eurasian magpie (EM), Eurasian tree sparrow (ETS), and house sparrow (HS).

The tables below display the full selection process in two sections:

1. **Random effects structure:** Comparison of alternative random-effects structures based on the lowest AICc.
2. **Fixed effects selection:** Ranking of candidate models (fixed-effects models) using the selected random structure. Inference is based on the confidence set of models accumulating ≥ 90% of Akaike weights (w_i).

The tables report parameter estimates (β ± SE) and their 95% confidence intervals [95% CI] derived from unconditional model averaging over the confidence set. Marginal (R^2m) and conditional (R^2c) coefficients of determination are reported for the top-ranked model in each analysis.

Table S1. Model selection and model-averaged coefficients for aggression rates between monk parakeets and native birds.

	MP ↔ RP	MP ↔ EM	MP ↔ ETS	MP ↔ HS
**Random effects structure**				
No random factors	285.59	255.43	20.37	25.07
Bait type	283.84	255.74	21.10	**23.43**
Interval	260.88	**228.61**	**19.86**	26.64
Park	287.52	254.27	21.06	27.10
Park / bait type	285.89	256.33	23.17	25.87
Park / interval	262.93	230.68	23.17	28.72
Park / interval / bait type	**243.78**	231.90	23.53	31.24
**Fixed effects selection**				
Null model	251.80 (0.01)	231.45 (0.05)	**16.75 (0.28)**	**21.04 (0.14)**
Directionality	249.06 (0.05)	**227.95 (0.26)**	**18.78 (0.10)**	**18.81 (0.44)**
N° of aggressors	255.71 (0.00)	**228.00 (0.25)**	**16.04 (0.39)**	**20.05 (0.24)**
Directionality + n° of aggressors	**245.59 (0.27)**	**228.02 (0.25)**	**17.77 (0.17)**	**21.05 (0.14)**
Directionality × n° of aggressors	**243.78 (0.67)**	**228.61 (0.19)**	19.86 (0.06)	23.43 (0.04)

R^2m / R^2c	0.02 / 1.00	0.01 / 0.96	0.04 / 1.00	0.35 / 1.00
**Model-averaged coefficients**				
Directionality (β ± SE)	0.73 ± 0.48 [-0.21, 1.67]	0.33 ± 0.31 [-0.28, 0.94]	5.47 ± 5180* [-10147, 10158]	2.86 ± 25.92* [-47.9, 53.7]
N° of aggressors (β ± SE)	**0.09 ± 0.03 [0.03, 0.15]**	0.09 ± 0.08 [-0.07, 0.25]	0.13 ± 0.22 [-0.30, 0.56]	0.16 ± 0.41 [-0.64, 0.96]
Directionality × n° of aggressors (β ± SE)	-0.05 ± 0.04 [-0.13, 0.03]	-0.02 ± 0.04 [-0.10, 0.06]	—	—

Note: In the "Fixed effects selection" section, values in parentheses indicate Akaike weights (w_i). In the "Model-averaged coefficients" section, values in brackets indicate the 95% confidence intervals [95% CI]. Bold text denotes the selected random-effects structure, the models included in the 90% confidence set, and the 95% CIs that do not span zero. The symbol "—" indicates that the term was not retained in the top model set used for averaging. Estimates marked with an asterisk (*) have extremely large standard errors due to extreme data scarcity (e.g., n=1 event for ETS, n=2 for HS) and should be interpreted with caution.

Table S2. Model selection and model-averaged coefficients for intimidation rates between monk parakeets and native birds.

	MP ↔ RP	MP ↔ EM	MP ↔ ETS	MP ↔ HS
**Random effects structure**				
No random factors	515.25	410.04	165.99	31.56
Bait type	**503.85**	408.25	**146.46**	33.86
Interval	514.37	**403.74**	167.70	**23.27**
Park	509.91	409.04	158.63	33.86
Park / bait type	505.79	410.14	148.55	36.23
Park / interval	510.56	405.60	160.68	36.23
Park / interval / bait type	512.31	404.76	162.74	31.31
**Fixed effects selection**				
Null model	519.77 (0.00)	458.08 (0.00)	317.64 (0.00)	37.11 (0.01)
Directionality	509.57 (0.03)	417.30 (0.00)	148.53 (0.09)	**18.74 (0.64)**
N° of intimidators	514.93 (0.00)	420.33 (0.00)	232.25 (0.00)	35.64 (0.02)
Directionality + n° of intimidators	**504.65 (0.39)**	**404.90 (0.36)**	**144.39 (0.67)**	**20.97 (0.25)**
Directionality × n° of intimidators	**503.85 (0.58)**	**403.74 (0.64)**	**146.46 (0.24)**	23.27 (0.08)
R^2m / R^2c	0.07 / 0.27	0.22 / 0.51	0.89 / 0.94	0.04 / 1.00
**Model-averaged coefficients**				
Directionality (β ± SE)	-0.25 ± 0.27 [-0.78, 0.28]	**0.93 ± 0.28 [0.38, 1.48]**	6.29 ± 137.5* [-263, 275]	10.30 ± 32.1* [-52.5, 73.1]
N° of intimidators (β ± SE)	0.03 ± 0.02 [-0.01, 0.07]	**0.16 ± 0.05 [0.06, 0.26]**	-0.39 ± 130.3* [-256, 255]	0.01 ± 0.23 [-0.44, 0.46]

Directionality × n° of intimidators (β ± SE)	-0.02 ± 0.02 [-0.06, 0.02]	-0.05 ± 0.05 [-0.15, 0.05]	0.49 ± 130.3* [-255, 256]	—

Note: In the "Fixed effects selection" section, values in parentheses indicate Akaike weights (w_i). In the "Model-averaged coefficients" section, values in brackets indicate the 95% confidence intervals [95% CI]. Bold text denotes the selected random-effects structure, the models included in the 90% confidence set, and the 95% CIs that do not span zero. The symbol "—" indicates that the term was not retained in the top model set used for averaging. Estimates marked with an asterisk (*) have extremely large standard errors due to data scarcity and should be interpreted with caution.
