# Supplementary material for: Balancing conflict and coexistence: Interactions between invasive monk parakeets and native urban birds
Source: Ecol Appl. 2026 Jun 18;36(4):e70275. doi: 10.1002/eap.70275 (PMC13276877; doi:10.1002/eap.70275)
Supplement: Supplementary file 6 — Appendix S6: [file EAP-36-e70275-s004.pdf]

Appendix S6

Balancing conflict and coexistence: Interactions between invasive monk parakeets and native urban birds

Jon Blanco-González, Isabel López-Rull, Fernando Enríquez and Luis Cayuela

Ecological Applications

Appendix S6: Model selection results for spatial abundance correlations and species-specific validation for *Passer* spp.

This appendix details the multimodel inference results analyzing the influence of the Kilometric Abundance Index (KAI) of monk parakeet nesting chambers—used as a proxy for parakeet abundance—on the KAI of sparrows and blackbirds across surveyed transects (**Table S1**).

The table below displays the full selection process in two sections:

1. **Random effects structure:** Comparison of alternative random-effects structures based on the lowest AICc.
2. **Fixed effects selection:** Ranking of candidate models (fixed-effects models) using the selected random structure. Inference is based on the confidence set of models accumulating ≥ 90% of Akaike weights (w_i).

The tables report parameter estimates (β ± SE) and their 95% confidence intervals [95% CI] derived from unconditional model averaging over the confidence set. Marginal (R^2m) and conditional (R^2c) coefficients of determination are reported for the top-ranked model in each analysis.

Species-specific validation for sparrows: In the main analyses, tree sparrows and house sparrows were pooled as *Passer* spp. due to inconsistent taxonomic differentiation during the 2021–2022 surveys. To assess whether this pooling could mask contrasting species-specific responses, we repeated the analysis using the subset of data from 2023, where both species were recorded separately (**Table S1**). The model-averaged 95% confidence intervals for the effect of nest chambers spanned zero across all three groups (tree sparrows, house sparrows, and pooled *Passer* spp.). Consequently, no statistically significant associations were detected between parakeet chamber density and the abundance of either sparrow species individually in 2023. This crucially indicates that pooling the species does not mask diametrically opposed significant responses (e.g., a strong positive association in one species vs. a strong avoidance in the other).

Furthermore, the lack of statistical significance in 2023 is largely attributable to a substantial reduction in statistical power caused by the management intervention. The intensive parakeet control program executed between 2021 and 2023 drastically compressed the upper end of the parakeet density gradient across the study area. Specifically, the maximum estimated KAI of

parakeet chambers dropped by 42% in 2023 compared to 2021. The absence of high-density parakeet sites in 2023—which act as critical leverage points in the regression models—severely limited our ability to detect spatial avoidance patterns that are clearly apparent when analyzing the full 2021–2023 gradient. Thus, the pooled analysis offers a valid approximation of the genus-level interaction trends within the studied context, as integrating data from all three years is essential to encompass the full invasion gradient and provide the necessary statistical power to detect negative spatial associations.

Table S1. Model selection and model-averaged coefficients for the Kilometric Abundance Index (KAI) of sparrows and blackbirds.

	KAI of *Passer* spp. (2021—2023)	KAI of black-birds (2021—2022)	KAI of *Passer* spp. (2023)	KAI of tree sparrows (2023)	KAI of house sparrows (2023)
**Random effects structure**					
No random factors	4038.89	2611.40	1275.66	924.35	810.44
Park	4017.95	2566.71	1265.57	896.55	788.01
Transect	3986.29	2583.84	**1259.61**	909.84	793.45
Park / transect	**3984.35**	**2565.98**	1261.54	**893.66**	**785.61**
**Fixed effects selection**					
Null model	3993.73 (0.00)	2580.41 (0.00)	**1259.15 (0.61)**	**892.12 (0.75)**	**785.70 (0.56)**
KAI of chambers	3991.76 (0.01)	2572.26 (0.01)	**1260.01 (0.39)**	**894.27 (0.25)**	**786.23 (0.44)**
Year	3988.05 (0.06)	2569.85 (0.04)	—	—	—
KAI of chambers + year	**3983.88 (0.51)**	**2564.39 (0.65)**	—	—	—
KAI of chambers * year	**3984.35 (0.41)**	**2565.98 (0.29)**	—	—	—
R^2m / R^2c	0.13 / 0.98	0.17 / 0.98	—	< 0.01 / 0.72	< 0.01 / 0.51
**Model-averaged coefficients**					
KAI of chambers (β ± SE)	**-0.08 ± 0.04 [-0.15, -0.02]**	**0.11 ± 0.04 [0.03, 0.19]**	-0.03 ± 0.05 [-0.13, 0.07]	0.01 ± 0.05 [-0.09, 0.10]	-0.06 ± 0.11 [-0.27, 0.14]
Year (β ± SE)					
2022 (vs 2021)	0.17 ± 0.09 [-0.01, 0.34]	**-0.20 ± 0.07 [-0.35, -0.05]**	—	—	—
2023 (vs 2021)	0.09 ± 0.07 [-0.05, 0.23]	—	—	—	—
KAI of chambers × Year (β ± SE)					
Year (2022) × KAI of chambers	-0.03 ± 0.04 [-0.10, 0.05]	0.01 ± 0.02 [-0.03, 0.04]	—	—	—

Year (2023) × KAI of chambers	0.00 ± 0.02<br>[-0.04, 0.04]	—	—	—	—

Note: In the "Fixed effects selection" section, values in parentheses indicate Akaike weights (w_i). In the "Model-averaged coefficients" section, values in brackets indicate the 95% confidence intervals [95% CI]. Bold text denotes the selected random-effects structure, the models included in the 90% confidence set, and the 95% CIs that do not span zero. The symbol "—" indicates parameters that are either not applicable (e.g., 'Year' in single-year analyses), not retained in the top model set, or where the null model was the top-ranked model (rendering R^2 values negligible or undefined).
