# Supplementary material for: Balancing conflict and coexistence: Interactions between invasive monk parakeets and native urban birds
Source: Ecol Appl. 2026 Jun 18;36(4):e70275. doi: 10.1002/eap.70275 (PMC13276877; doi:10.1002/eap.70275)
Supplement: Supplementary file 7 — Appendix S7: [file EAP-36-e70275-s006.pdf]

Appendix S7

Balancing conflict and coexistence: Interactions between invasive monk parakeets and native urban birds

Jon Blanco-González, Isabel López-Rull, Fernando Enríquez and Luis Cayuela

Ecological Applications

Appendix S7: Abundances of native tenants

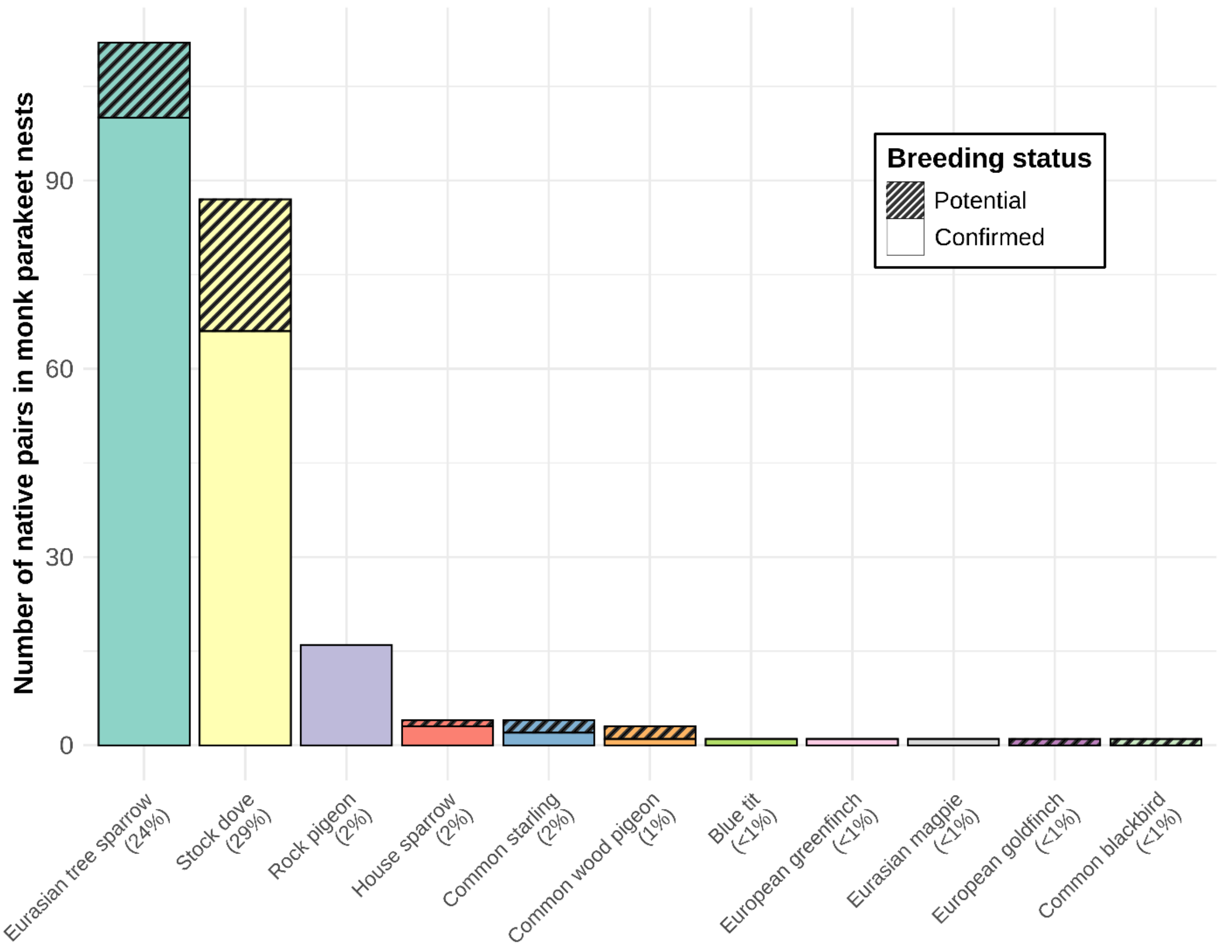

Figure S1. Abundances of native bird species nesting in monk parakeet nests. Solid-color regions indicate confirmed breeding pairs (highly probable breeding status), while striped regions indicate potential breeders (ambiguous breeding status). Among the 11 native species observed, confirmed breeding was recorded for all except the European goldfinch *Carduelis carduelis* and the common blackbird *Turdus merula*. The percentages shown next to each species name represent the proportion of nests in which at least one pair of that species was found breeding, including both confirmed and potential cases.
